# 

**Published:** 1901-10

**Authors:** 


### ASSOCIATION MEETINGS.

OCTOBER.

### MAINE VETERINARY MEDICAL ASSOCIATION.

Next regular meeting at Lewiston, October 9th.

F. E. FREEMAN,
ROCKLAND, ME. Secretary.

### MISSOURI VETERINARY MEDICAL ASSOCIATION.

THE tenth annual meeting of the Missouri State Veterinary Association will be held in Kansas City, October 22d and 23d.

October 22d, 10 A M. Business meeting.

1 P.M. Papers: "An Enzoötic Attack of Chorea Among Cattle," Dr. A. D. Knowles; "Sclerastoma Tetracanthus of the Horse," Dr. W. E. Martin; "Pleurisy of the Horse," Dr. F. F. Brown; "My Practical Experience Since our Last Meeting," Dr. T. M. Klutts; subject not given, Dr. E. Brainard.

7.30 P.M. Papers: "Immunizing Northern-bred Cattle Against the Southern Cattle Fever," Dr. J. W. Connaway; "Acute Rheumatism of the Horse," Dr. T. J. Menestrina; "Azoturia," Dr. R. H. Thomas; "Tuberculosis of Cattle and Swine," Dr. W. R. Cooper; "Hog-cholera and Swine-plague," Dr. A. Long; "Actinomycosis," Dr. H. H. George.

October 23, 8 A.M. Surgical and Dental Clinic.

1 P.M. Pathological Exhibit.

3 P.M. A visit to the Cattle-show and sales.

8 P.M. A visit to the Kansas City Horse-show.

B. F. KNAPP,
3725 MICHIGAN AVENUE, KANSAS CITY, MO. Secretary.

### NEBRASKA STATE VETERINARY ASSOCIATION.

Early in October at Omaha. A. T. PETERS,
LINCOLN, NEB. Secretary.

NOVEMBER.

### TENNESSEE VETERINARY MEDICAL ASSOCIATION.

Next meeting at Nashville, about the 10th.

JOSEPH PLASKETT,
NASHVILLE, TENN. Secretary.

### CORRECTION.

DEATH OF DR. SHAUB. In the September issue of the JOURNAL, in referring to the death of Dr. A. E. Shaub, of Lancaster, Pa., we inadvertently associated the ownership of the veterinary hospital and practice of Dr. J. C. Shaub, which led some of the latter's friends and acquaintances to think he was the one who had died.

### PERSONALS.

Dr. B. W. Gheen, formerly of Washington, D. C., is now a resident of Greenville, Miss. Plantation practice is a feature of his present location.

Dr. J. M. Doran, of Barry, Ill., is a breeder of horses in connection with his veterinary practice.

Dr. T. F. Mullaney has just been unanimously reappointed city veterinarian of Kansas City, Mo. This is Dr. Mullaney's third term.

Dr. Joseph W. Parker, inspector, has been transferred from Del Rio, Texas, to San Antonio, in the same State. His work is along the quarantine line and in sheep inspection.

Dr. B. F. Senseman, of Northwest Philadelphia, is an active member of the Road Drivers' Association of Philadelphia.

Dr. Thomas Castor, V.M.D., graduate of the University of Pennsylvania, has been transferred from San Antonio, Texas, to Trinidad, Colorado. He succeeds the late Dr. A. M. Rook in the inspection of sheep for New Mexico and Colorado.

Dr. H. H. Weinberg, of Philadelphia, devotes his leisure hours to following the uncertainties of his fast pacing mare at the matinee races of the gentlemen's road clubs.

Dr. W. D. Savage, of Zanesville, Ohio, died early in the spring.

Dr. John W. Adams, of Philadelphia, was a June visitor to the Pan-American Exposition at Buffalo.

Dr. Joseph J. Smith, of Albany, Oregon, reports practice unusually good in that State. Only graduates are allowed to practice in Oregon.

Dr. Jacob N. Becker, of Palmyra, Pa., called at the JOURNAL office the middle of September. He reports that Lebanon County is devastated by hog-cholera.

Dr. J. Massie, of Kingston, Canada, a member of the Royal Artillery, is also a graduate in pharmacy, and has conducted a drug store for more than twelve years.

Dr. Joseph W. Parker, U. S. Inspector, Bureau of Animal Industry, has been transferred to San Antonio, Tex.

Dr. J. C. Shaub, of Lancaster, Pa., spent some time on a trip to Labrador.

Dr. Thomas Trinder, of Charlotte, N. C., will take a post-graduate course the coming winter at the McKillip Veterinary College, Chicago. Dr. Trinder is a graduate of the Ontario Veterinary College.

Dr. D. W. Patton, Bureau of Animal Industry, has been transferred from South Omaha, Neb., to Union Stock-yards, Chicago, Ill.

Dr. H. Buss, of St. Johnsbury, Vt., has removed to Deansboro, N. Y.

Dr. G. W. Brallier, of Berlin, Pa., is not a follower of the veterinary profession any longer, but gives his time to the conducting of a pharmacy.

Drs. J. O. George, of Camden, and R. W. Hewitt, of the Bureau of Animal Industry, were visitors to the JOURNAL office in September.

Dr. Arthur M. Rork, who recently died at Pueblo, Colo., was a graduate of the Chicago Veterinary College, class of '93. Dr. Rork died just ten days after Dr. F. T. Shannon, of the Bureau, both deaths resulting from typhoid fever. Dr. Shannon was located at Monet, Mo., before being transferred to Pueblo.

Dr. Friedheim, of Rock Hill, S. C., will temporarily succeed Dr. Thomas Trinder at Charlotte, N. C., while the latter enters upon a post-graduate course at one of the colleges.

The young son of Dr. T. F. Mullaney, but five years of age, was an active orator in the Bryan campaign of 1900, and was taken through many of the cities and towns of the West during that period.

The New Haven Veterinary Sanitarium, of New Haven, Conn., is conducted by Dr. F. G. Atwood, a graduate of the Ontario Veterinary College and post-graduate of Johns Hopkins Medical College.

Dr. J. W. Fink, of the Bureau of Animal Industry, is now stationed at Buffalo, N. Y., attending the government exhibit at the Pan-American Exposition.

Dr. W. H. Pendry, of Brooklyn, N. Y., was unable to attend the meeting at Atlantic City, owing to the needs and demands on his time incidental to a prospective political career.

